# Evaluation and deployment of a unified MPPT controller for hybrid Luo converter in combined PV and wind energy systems

**DOI:** 10.1038/s41598-024-53605-z

**Published:** 2024-02-08

**Authors:** K. Kumar, V. Lakshmi Devi, C. Dhanamjayulu, Hossam Kotb, Ali ELrashidi

**Affiliations:** 1 Department of Electrical and Electronic Engineering, Sri Venkateswara College of Engineering, Tirupati, India; 2 School of Electrical Engineering, Vellore Institute of Technology, Vellore, Tamil Nadu India; 3 Department of Electrical Power and Machines, Faculty of Engineering, Alexandria University, Alexandria, 21544 Egypt; 4 Electrical Engineering Department, University of Business and Technology, Ar Rawdah, 23435 Jeddah, Saudi Arabia; 5 Engineering Mathematics Department, Faculty of Engineering, Alexandria University, Alexandria, 21544 Egypt

**Keywords:** Energy science and technology, Engineering

## Abstract

This work emphasizes the development and examination of a Hybrid Luo Converter integrated with a unified Maximum Power Point Tracking (MPPT) for both grid and independent hybrid systems. The primary objectives of this hybrid system are to efficiently harness power from intermittent and variable renewable sources while elevating low-voltage energy inputs to utility-grade levels. Unlike previous studies employing specific MPPT algorithms for solar and wind sources, this work aims to simplify the control system by utilizing a unified MPPT controller. This research also introduces a novel approach involving dual-lift hybrid Luo converters to create hybrid systems, operating exclusively or concurrently based on the availability of renewable resources. To maximize power generation from all renewable sources, a unified MPPT algorithm is developed. The hybrid system, incorporates 500 W wind and 560 W PV systems, the innovative Luo converter, and the unified MPPT controller. A comprehensive comparative analysis is presented, comparing the hybrid system's performance with that of traditional control algorithms, such as the Perturb & Observe, and Radial Basis Function Network controllers. The successful prototype of the converter validates the practicality of the proposed approach.

## Introduction

In recent decades, the usage of fossil fuels has drastically augmented owing to the mandate for electricity in human day-to-day life^1,2^. The continued consumption of fossil fuels has led to their depletion, and their combustion produces harmful by-products. Finding the best clean and green energy sources that can keep up with the needs of today's fully electrified society has been a major focus of scientific inquiry for the past few decades^3,4^. Photovoltaic (PV) and wind are two of the utmost promising clean energy in use today because of their low or no environmental impact and high potential for widespread adoption. The grid installed capacity of the renewable energy sources is 133,886.18 MW, in which PV contribution is 54% and wind contribution is 33% as of the 31st December 2023^5^.

The unpredictable and at times unpredictable input weather conditions of clean energy sources make for erratic power output and an inability to keep up with load demands^6^. This raises doubts about harnessing the power of renewable. The solution to the aforesaid issues, the incorporation of multiple Regenerative sources with storage devices is one of the most promising solutions. Integration methodologies and maximum power tracking power converters are needed to maximize hybrid system power. The literature contains many power converters for hybridizing energy sources, including AC shunt, DC shunt, and hybrid mixed systems.

In^7^, the authors developed a high-gain converter for multiple RES. By changing the linked inductor winding turns ratio, the system achieves high voltage transfer gain. The experimental findings are compared with the theoretical values and the implanted hybrid system provides an overall efficacy of 94%.

A novel DC-DC converter of three input ports has been formulated and presented in^8^, the configuration provides input terminals for solar power and Fuel Cell systems and a bi-directional port for connecting the battery system. Four peculiar duty ratio signals are generated to govern the power switches in the developed configuration.

A new zero voltage switching converter was presented in^9^, a multi-input converter for boosting the peculiar low voltage to desired and stable voltages. A complementary circuit consisting of negligible inductance is engaged to turn on the converter in two modes.

A flexible matrix converter was invented by^10^ to add renewable energy sources to the grid. The nine-switch matrix converter works similarly, although it has additional power inputs. The lower section unites three hybrid energy systems, while the above section communicates with the wind power generator. Simple Ex-OR gate logic modulates and controls the system, which features a top half that operates as a 3-Ø rectifier for the wind and a bottom half that provides three sources with DC-DC conversion.

The authors^11^ developed an integrated Cuk-SEPIC converter by sharing the Cuk inductor L_2_ with the SEPIC and by rearranging the converter diodes which reduces the converter components for the hybrid system and eliminates the need for the input filter.

The additional major issue is the requirement of individual and dedicated maximum power tracking algorithms, which causes complexity in the system implementation. In literature, many authors have proposed universal MPPT controllers^12–16^, which are worm to elicit the maximal power from RES, but the universal MPPT techniques have limitations of requiring a dedicated controller for each source, which in turn increases the implementation complexity.

MPPT controllers vary in power extraction, tracking speed, and other features. Authors^17–21^ suggested maximum power point (MPP) extraction from RES with consistent tracking speed and MPPT controller characteristics to solve system implementation issues. Many MPPT algorithms remove MPP from PV and wind sources^22–26^.

A hybrid Luo (HL) converter with one MPPT controller is shown in this study. The suggested converter splits charging and DC link capacitors across converters with negative output to produce a multi-input system. The solar-wind energy system may now harvest maximum power points with a unified MPPT controller. A hybrid converter MPPT architecture controls power from both sources better.

In this article, "Design of hybrid system" section presents the design of a proposed hybrid system. In "Analysis of hybrid Luo converter" section, the analysis of the hybrid Luo converter. The Unified MPPT control techniques are discussed in "Unified MPPT control techniques" section. The analysis and discussion of the proposed system are presented in "Analysis and discussion on the proposed system" section. Finally, "Conclusion" section presents the conclusion.

## Design of hybrid system

**Figure 1:**
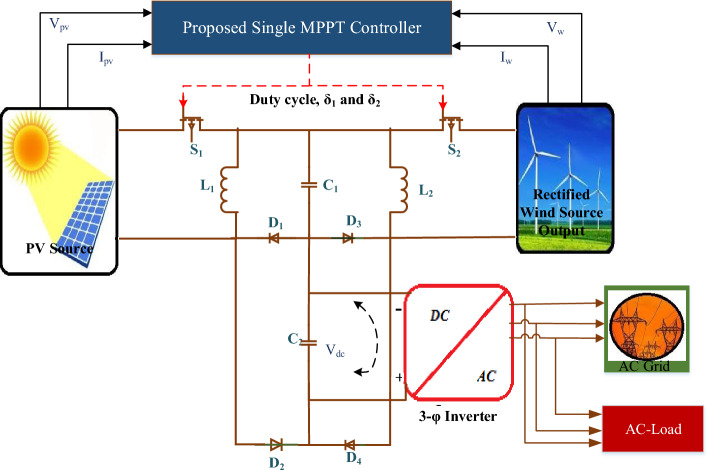
Proposed hybrid system with HL converter and unified MPPT topology.

Figure 1 depicts the system with a unified MPPT controller. This system has a Luo DC-DC converter and a controller for MPP extraction from a hybrid RES system.

### PV system

**Figure 2:**
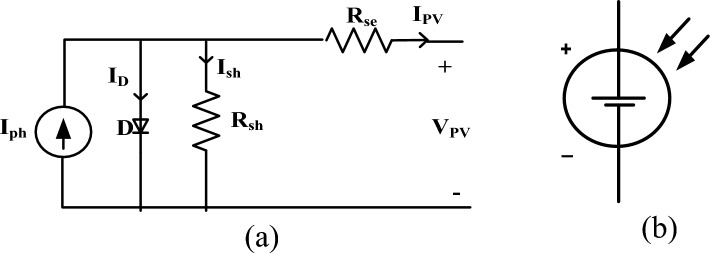
(**a**) PV cell, (**b**) symbolic PV cell representation.

PV arrays have series and parallel modules. Figure 2 shows the PV cell circuit and symbol.

The PV system's output is determined by the PV module's specification parameters. In the developed system, the BP solar SX3190 PV module is designated to model a 560W system. Table 1 lists the specific parameters and the system's production is dependent on temperature and solar radiation levels.

**Table 1:** Details of 560 PV system.

Details	Specifications
Max. voltage V_MP_	24.3 V
Max. current I_MP_	7.829 A
Max. power P_MP_	560 W
Solar radiation G	1000 W/m^2^
Temp. T	25 °C
SC current I_SC_	8.51 A
OC voltage V_oc_	30.6 V
Series modules/string	1
Parallel strings	3

### Wind system

A converter is placed across a wind turbine's generator and the electrical grid or load to optimize wind power. The received power from the system is expressed by Eq. (1)^18^.

1 $$P_{m} = \frac{1}{2}\rho AV_{v}^{3}$$

The generated mechanical power is dependent on the wind turbine friction coefficient, tip speed ratio, air density, and wind velocity, according to Eq. (1). The Aeolos-H 500 W model's default settings are utilized to construct a 500 W wind system for use in a hybrid setup. Details are provided in Table 2.

**Table 2:** Details of a wind system.

Details	Specifications
Type	PMSG
Power rating P	500 W
Wind speed-cut-in V_d_	4 m/s
Wind speed-rated V_n_	12 m/s
Pair of poles P_P_	2
Torque/current T/A	1.1216 N m/A
Co-efficient of friction B	0
Impedance R_a_	0.775 Ω
Magnetizing flux Ø_m_	0.37387 wb
Inductance L_q_ and L_d_	7.31 mH

## Analysis of hybrid Luo converter

A hybrid Luo converter topology is derived in this section for the amalgamation of renewable PV and wind sources with reduced power converter switches and converter components count.

### Design of converter

**Figure 3:**
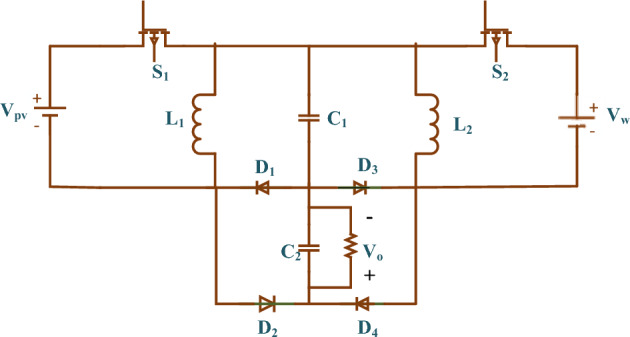
HL converter topology.

The hybrid Luo (HL) converter in Fig. 3 is based on the super lift Luo converter^27^.

Negative-output super lift two solar and wind input ports are created by merging super lift Luo converters. In Fig. 3, the charging and capacitors of C_1_ and C_2_ are divided to provide the mutually merged configuration. depending on renewable energy sources, the suggested converter should manage four circumstances, which may be attained by delivering the pulse to the control switches of separate dual input converters and interpreted in the following sections.

### Operating conditions of HL converter

#### Condition-1: S_1_—closed, S_2_—closed

**Figure 4:**
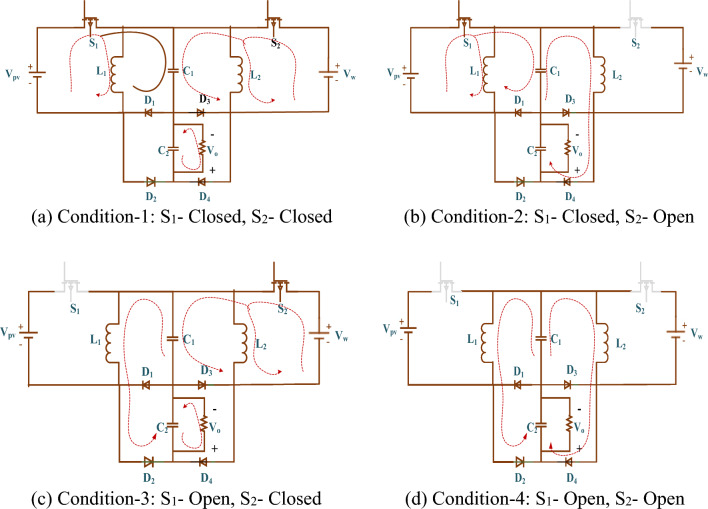
(**a**–**d**) Operating conditions of proposed HL converter.

S_1_ and S_2_ are closed in condition 1. Figure 4a shows the sharing capacitor C_1_ charging with V_PV_ and V_W_ voltage. Equation (2) calculates the inductor currents i_L1_ and i_L2_.

2 $$i_{L1} = I_{w} + \frac{{V_{w} + V_{c1} }}{{L{}_{1}}}t\;\;{\text{and}}\;\;i_{{{\text{L}}2}} = {\text{I}}_{{{\text{pv}}}} + \frac{{{\text{V}}_{{{\text{pv}}}} + {\text{V}}_{{{\text{c}}1}} }}{{{\text{L}}{}_{2}}}{\text{t}}$$

#### Condition-2: S_1_—closed, S_2_—open

Condition-2 has S_1_ closed and S_2_ open. In Fig. 4b, V_W_ charges C_1_'s sharing capacitor. Equation (3) calculates the inductor currents i_L1_ and i_L2_.

3 $$i_{L1} = I_{w} + \frac{{V_{w} + V_{c1} }}{{L{}_{1}}}t\;\;{\text{and}}\;\;i_{L2} = I_{dc} - \frac{{V_{c1} }}{{L{}_{2}}}t$$

#### Condition-3: S_1_—open, S_2_—closed

Condition-3 has S_1_ open and S_2_ closed as in Fig. 4c, the sharing capacitor C1 is charging through V_PV_. Equation (4) calculates the inductor currents i_L1_ and i_L2_.

4 $$i_{L1} = I_{dc} - \frac{{V_{c1} }}{{L{}_{1}}}t\;\;{\text{and}}\;\;i_{L2} = I_{pv} + \frac{{V_{pv} + V_{c1} }}{{L{}_{2}}}t$$

#### Condition-4: S_1_—open, S_2_—open

The switches S_1_ and S_2_, are in the open position in condition 4. In this state, the DC link capacitor C2 is being discharged by the distribution capacitor and inductors L_1_ and L_2_, which results in the boosted voltage at the load side as shown in Fig. 4d. Equation (5) formulates the currents i_L1_ and i_L2_ over L_1_ and L_2_.

5 $$i_{L1} = I_{dc} - \frac{{V_{c1} }}{{L{}_{1}}}t\;\;{\text{and}}\;\;i_{L2} = I_{dc} - \frac{{V_{c1} }}{{L{}_{2}}}t$$

Table 3 is a list of the operating condition summaries for the proposed HL converter with switching positions.

**Table 3:** Operating conditions of HL converter.

Condition	Switch position		Diode operation				Inductor charge and discharge	
	S_1_	S_2_	D_1_	D_2_	D_3_	D_4_	L_1_	L_2_
1	Closed	Closed	FCS	RBS	FCS	RBS	V_W_	V_PV_
2	Closed	Open	FCS	RBS	RBS	FCS	V_W_	Capacitor, C_1_
3	Open	Closed	RBS	FCS	FCS	RBS	Capacitor, C_1_	V_PV_
4	Open	Open	RBS	FCS	RBS	FCS	Capacitor, C_1_	Capacitor, C_1_

*FCS* forward conduction state, *RBS* reverse blocking state.

**Figure 5:**
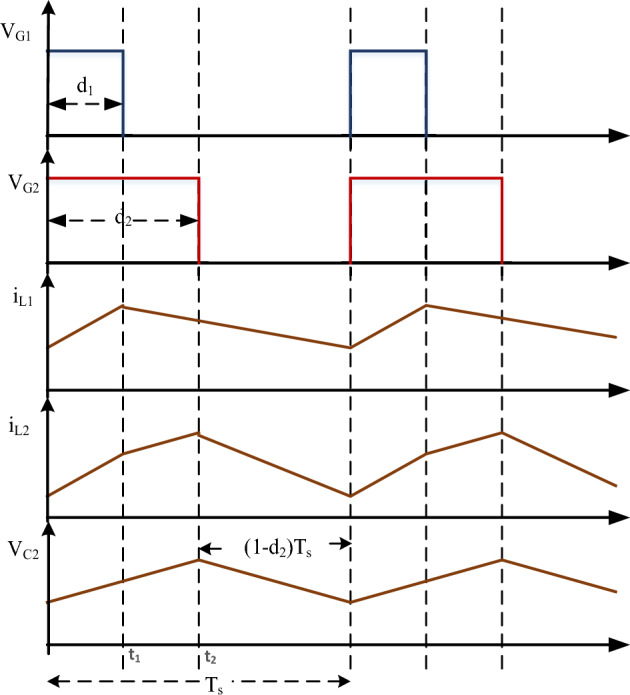
Proposed HL converter switching waveforms.

To provide hybrid multi-input power converters with fewer power converter components and to lessen the switching stress of the converter, the HL converter configuration is built by reordering and sharing the converter components between the converters. The proposed converter switching waveforms are shown in Fig. 5.

## Unified MPPT control techniques

The work aims to extract MPP from dynamically varying RES via maximum power tracking. P&O, Hill climbing, artificial neural networks, fuzzy logic controllers and bio-inspired algorithms are available for PV and wind. Due to its ease of use and predefined step size, the P&O approach is the most popular. Fuzzy and ANN-based MPPT can manage non-linearity.

### Unified P&O MPPT controller

A unified algorithm from the classic P&O controller generates a duty cycle for the 560 W HL converter-fed hybrid PV and 500 W wind systems.

Equation (6) calculates PV output power.

6 $${\text{P}}_{{{\text{PV}}}} = {\text{ V}}_{{{\text{PV}}}} *{\text{ I}}_{{{\text{PV}}}}$$

Equation (7) shows the DC link voltage-PV output voltage correlation.

7 $$V_{PV} = \left( {\frac{1}{1 - D}} \right)V_{dc}$$

Equation (8) calculates wind output power as

8 $${\text{P}}_{{\text{W}}} = {\text{ V}}_{{\text{W}}} *{\text{ I}}_{{\text{W}}}$$

The voltage at the DC link and solar power generation voltages are related in Eq. (9).

9 $$V_{W} = \left( {\frac{1}{1 - D}} \right)V_{dc}$$

**Figure 6:**
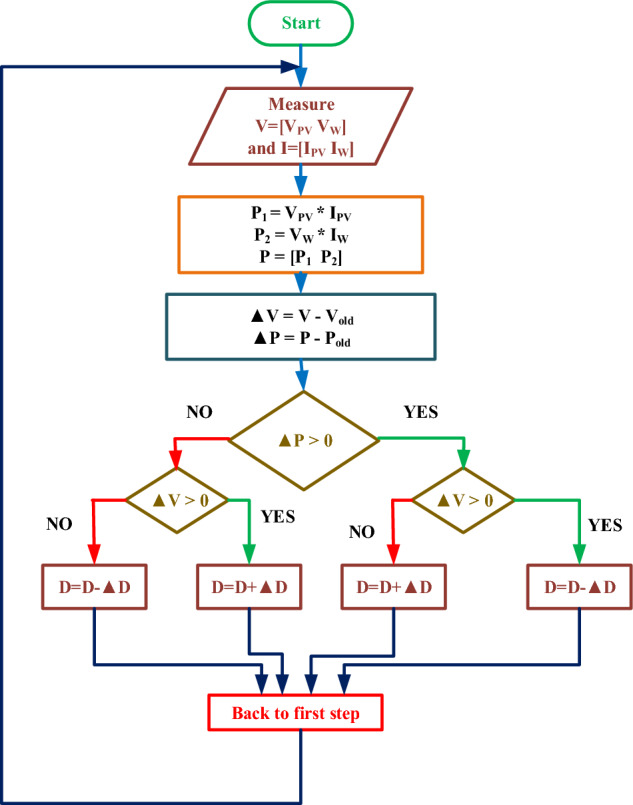
Modified P&O MPPT.

The suggested unified algorithm extracts maximum power from renewable sources in steps. Based on irradiance and velocity, the unified P&O MPPT approach delivers a hybrid system duty cycle. P&O-based control strategies require many assessments, slowing convergence is shown in Fig. 6. Comparing unified MPPT controllers to individual MPPT controllers, the latter provides a more straightforward and economical solution for renewable energy systems. Through full utilization of renewable energy sources, they minimize expenses, simplify system architecture, and enhance overall performance. Unified controllers are a good option for many installations due to their improved coordination, ease of monitoring and maintenance, and space savings; nevertheless, the choice ultimately depends on the particular system requirements and design considerations.

### RBFN MPPT controller

**Figure 7:**
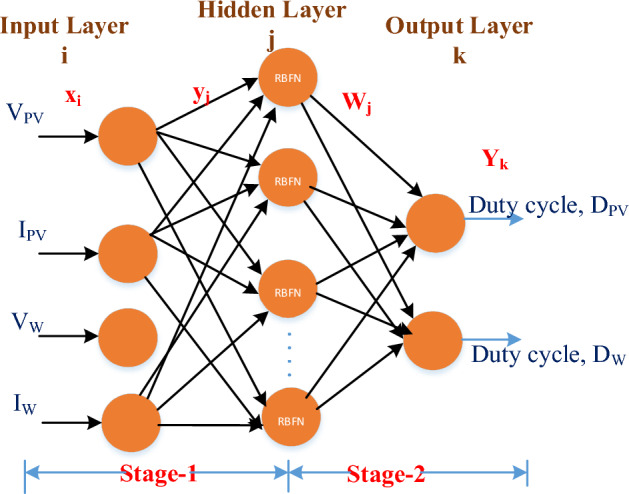
RBFN Unified MPPT controller.

The current section develops a unified ANN-based controller to monitor wind and PV maximum power points. Figure 7 exhibits an RBFN-based network featuring four input variables (V_PV_, I_PV_, V_W_, and I_W_), 529 concealed neurons in the hidden layer, and two regulatory switch duty cycles. In three steps, the same RBFN-based MPPT controller generates a duty cycle for concurrent renewable energy sources^17,18^. The nodal equations for RBFN-based single MPPT controllers are as follows:

#### Input layer

Input layer nodes immediately send V_PV_, I_PV_, V_W_, and I_W_ data to the next neuron layer. Equations (10) and (11) inure the input layer and total input.

10 $$x_{i}^{\left( I \right)} \left( n \right) = net_{i}^{\left( I \right)}$$

11 $$y_{i}^{\left( I \right)} \left( n \right) = f_{i}^{\left( I \right)} \left( {net_{i}^{I} \left( n \right)} \right) = net_{i}^{\left( I \right)} \left( n \right),^{{}} \quad i = 1,2,3,4$$

#### Hidden layer

The membership function for each hidden layer is Gaussian. Equation (12) accounts for all inputs, while Eq. (13) accounts for all outputs.

12 $$net_{j}^{\left( H \right)} \left( n \right) = - \left( {X - M_{j} } \right)^{T} \mathop \sum \limits_{j} \left( {X - M_{j} } \right)$$

13 $$\begin{aligned} y_{j}^{\left( H \right)} \left( n \right) & = f_{j}^{\left( H \right)} \left( {net_{j}^{\left( H \right)} \left( n \right)} \right) \hfill \\ \mathop {}\nolimits_{{}} \mathop {}\nolimits_{{}} \mathop {}\nolimits_{{}}^{{}} & = \exp \left( {net_{j}^{\left( H \right)} \left( n \right)} \right),\mathop {}\nolimits_{{}} j = 1,2,3,4 \hfill \\ \end{aligned}$$

#### Output layer

The controller's total output is made up of two neurons in output. These neurons use the linear activation function to create two diverse pulses, D_pv_ and D_w_. In Eqs. (14) and (15), we find the output layer, the total input, and the output.

14 $$net_{k}^{\left( O \right)} \left( n \right) = \sum\limits_{j} {w_{j} y_{j}^{\left( H \right)} \left( n \right)}$$

15 $$y_{k}^{\left( O \right)} \left( n \right) = f_{k}^{\left( O \right)} \left( {net_{k}^{\left( O \right)} \left( n \right)} \right) = net_{k}^{\left( O \right)} \left( n \right)$$

where, $x_{i}^{\left( I \right)}$ = input layer;$net_{i}^{\left( I \right)} \left( n \right)$ = input layer summated value; $net_{i}^{\left( H \right)} \left( n \right)$ = hidden layer summated value; $net_{i}^{\left( O \right)} \left( n \right)$ = output layer summated value; W_j_ = hidden and output layer connecting weight; M_j_ = output layer’s Mean deviation; $\sum\limits_{j} {}$ = standard deviation of the output layer.

Currents and voltages of PV and Wind regulate the system outputs, Solar photovoltaic (PV) irradiance and wind speed define each system's duty cycle. The Radial Basis Function Neural Network (RBFN) MPPT controller offers potential advantages over the Perturb and Observe (P&O) MPPT controller, including better adaptability to changing conditions, faster response, reduced oscillations, improved performance under partial shading, enhanced robustness, and the potential for online adaptation. The choice between them depends on factors like implementation details, cost, and real-time processing requirements.

## Analysis and discussion on the proposed system

**Figure 8:**
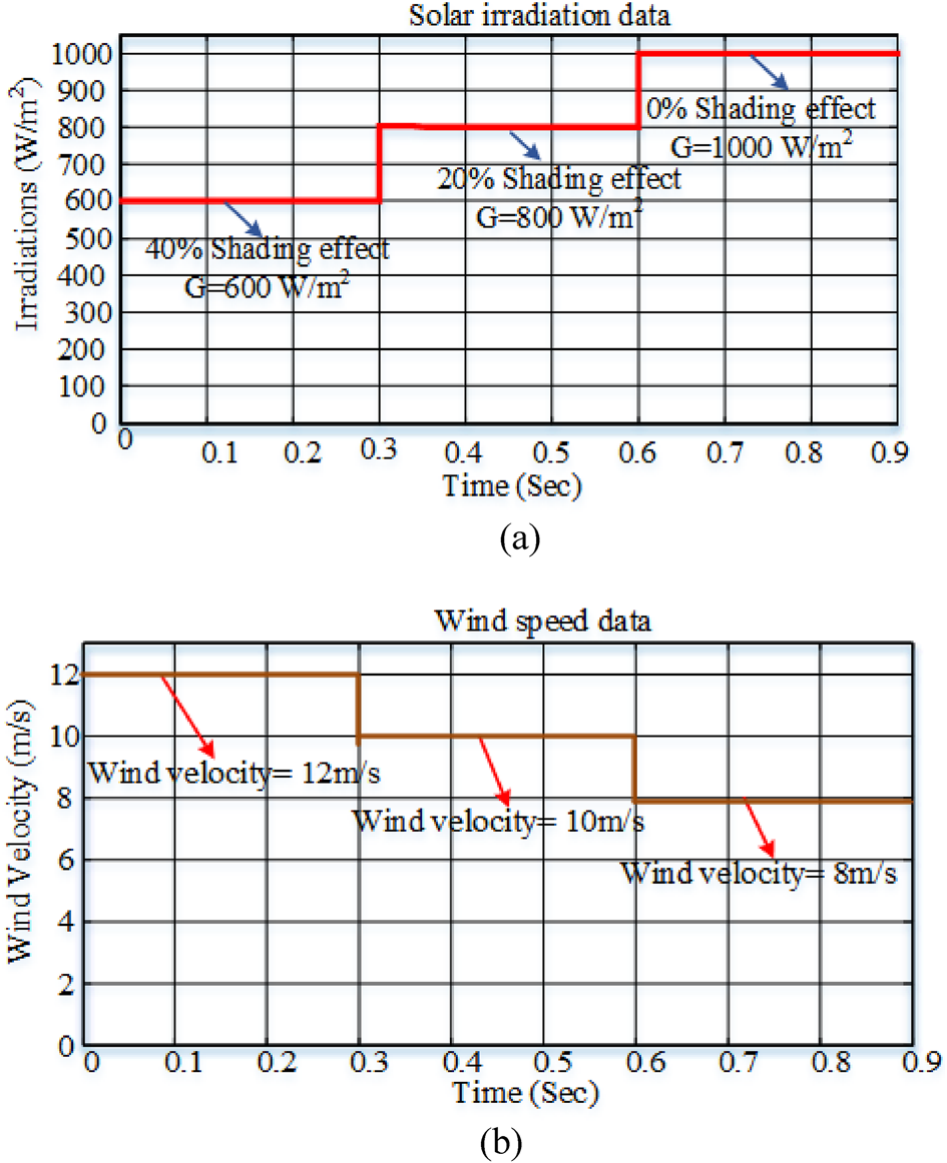
(**a**) Considered solar irradiation, (**b**) considered wind speed.

MATLAB/Simulink verifies HL converter topology performance. From the literature, PV and wind energies are contradictory, so to test the converter's feasibility in both exclusive and simultaneous modes, renewable sources are selected for 0–0.3 s, with the wind at 12 m/s and PV at 600 W/m^2^. As illustrated in Fig. 8, a wind velocity of 10 m/s with 800 W/m^2^ PV radiation and a velocity of the wind of 8 m/s with 1000 W/m^2^ are used for the 0.3–0.6 s and 0.6–0.9 s intervals, respectively.

The proposed hybrid system with a PV power rating of 560 W and wind power rating of 500 W with the developed converter topology detailed particulars are presented in Table 4.

**Table 4:** Detailed particulars of developed hybrid system.

Items/description	Specifications
Input voltage rating	V_PV_ = 24 V
	V_W_ = 24 V
Power rating	P_PV_ = 560 W
	P_W_ = 500 W
Switching frequency	f_s_ = 20 kHz
Inductor ratings	L_1_ = 3e^−3^ H
	L_2_ = 3e^−3^ H
Capacitor ratings	C_1_ = 1e^−3^ F
	C_2_ = 5e^−3^ F
Load resistance	R = 106 Ω

The hybrid system with the designed HL converter and the planned unified MPPT controllers is tested in standalone and grid-connected settings with three load zones based on renewable sources.

### Standalone mode

**Figure 9:**
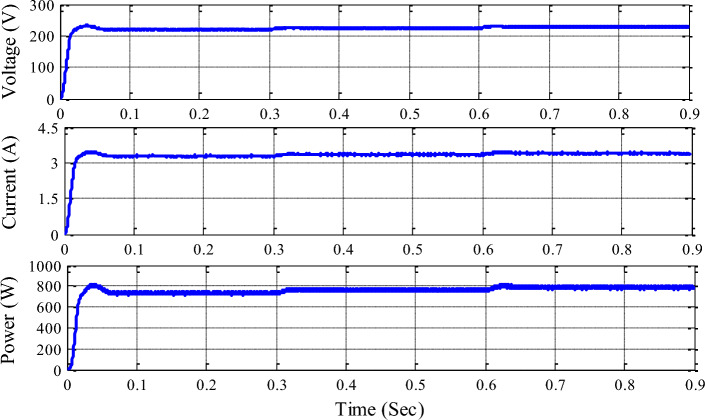
Voltage, current, and power at DC link capacitor with single P&O MPPT.

Figure 9 illustrates the functionality of the unified P&O MPPT architecture to demonstrate the voltage, current, and power measured at the DC link capacitor using the unified technique applied to the system. Due to the non-linear features of PV irradiance and Wind speed, the P&O approach is unable to collect the maximum amount of electricity from the sun and wind.

**Figure 10:**
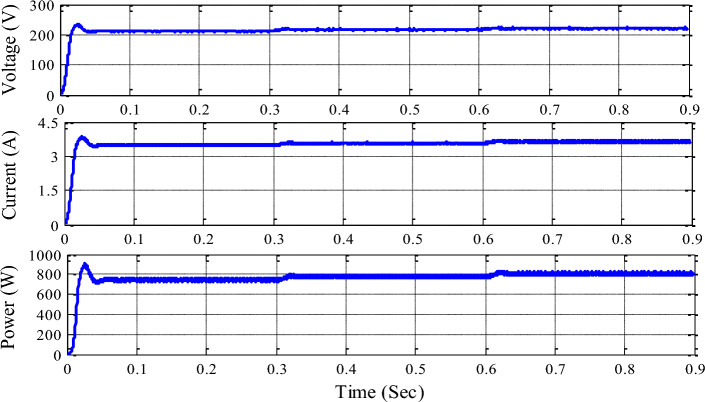
Voltage, current and power at DC link capacitor with unified RBFN MPPT.

Figure 10 demonstrates the voltage, current, and power measured at the DC link capacitor with a unified RBFN-based MPPT topology. RBFN-MPPT measures maximum power and stabilizes during irradiance and wind speed parameter changes. RBFN-based MPPT achieves constant DC link voltage due to its faster convergence speed. DC microgrid efficiency relies on stable DC link voltage.

Table 5 summarizes the hybrid system with updated MPPT control approaches and renewable sources. P&O MPPT generates 754.2 W and RBFN-based MPPT generates 756.7 W for 600 W/m2 and 12 m/s input data from 0 to 0.3 s. For 0.3 to 0.6 s, P&O MPPT develops 778 W and RBFN-based MPPT develops 781.2 W. For 0.6 to 0.9 s, P&O develops 801.2 W and RBFN develops 804.6 W. The table shows that the unified RBFN controller outperforms the unified P&O controller.

**Table 5:** The summary hybrid system.

Hybrid system with HL converter average output power			
Period (s)	0 to 0.3	0.3 to 0.6	0.6 to 0.9
PV radiation (W/m^2^)	600	800	1000
Velocity of the wind (m/s)	12	10	8
Unified P&O-MPPT	754.2 W	778.0 W	801.2 W
Unified RBFN-MPPT	756.7 W	781.2 W	804.6 W

### Experimental results

**Figure 11:**
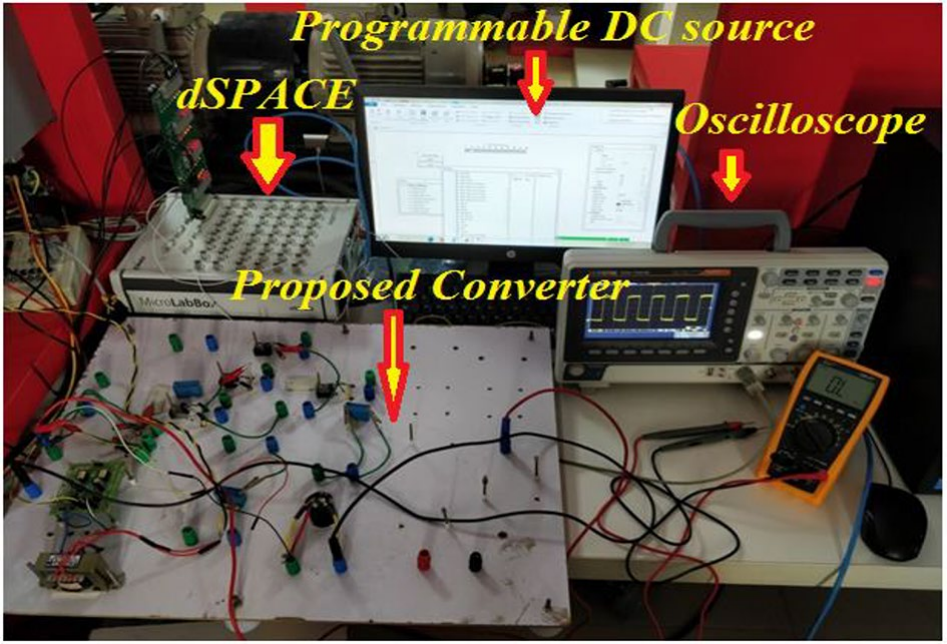
Experimental prototype.

To validate the contemplated converter, A laboratory-tested prototype model is developed for the scale-down model and it is depicted in Fig. 11.

MSOs evaluate the outcomes of experiments. Sensors for voltage (LV 20P) and current (LA 25N) from the dSPACE 1104 model allow for feedback control signals from a renewable source.

**Figure 12:**
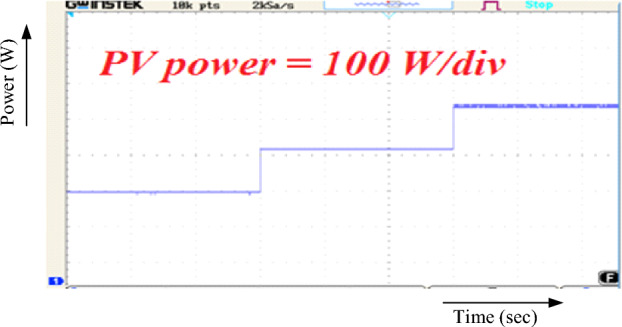
PV output power.

The PV output power of the converter arrangement is depicted in Fig. 12. The non-linear performance of the system is put to the test by the programmable DC Source's sudden shift in irradiance and wind speed. The most power may be produced from PV irradiation using RBFN-based MPPT.

**Figure 13:**
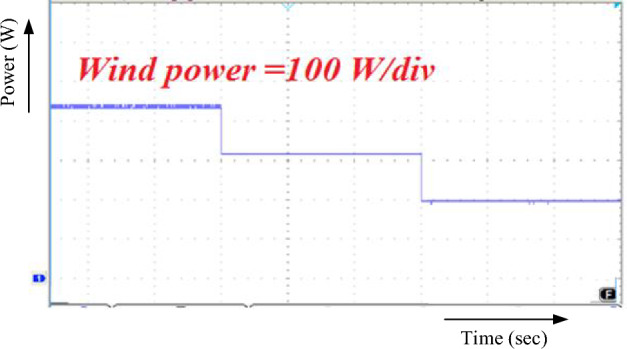
Wind output power.

Power generated by the wind with this converter setup is depicted in Fig. 13. The recommended layout is supported by the observed step change in the wind. The speed is 12-10-8 m/s. Energy output depends on wind speed, with 453.5 W, 377.7 W, and 299.3 W being generated at various gusts. As a result, MPPT based on RBFN efficiently measures wind velocity.

**Figure 14:**
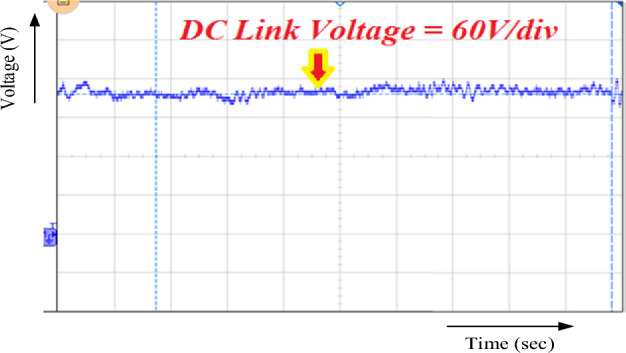
DC link output voltage.

Figure 14 illustrates the anticipated system DC link voltage. The hybrid system balances a constant (228 V) DC link voltage to sustain the DC microgrid. The constant DC connection voltage is the foundation of the RBFN unified MPPT technique.

**Figure 15:**
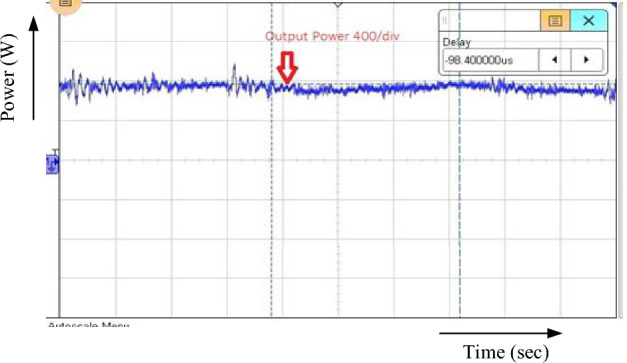
Output power.

Figure 15 displays the hybrid system's overall output power. The inputs of (503.4 w) and (299.2 w) of PV and Wind, respectively, yield the maximum power of (802.6 W).

### Grid mode

A MATLAB model of a 560W PV and a 500W wind, along with a suggested HL converter and a unified MPPT controller, are developed in MATLAB with an AC grid that has a rating of 230 V and 50HZ. This is done to test the hybrid system when it is linked to the grid. The following is an analysis of the load that is coupled to the proposed grid-connected system: for the first period, 0 to 0.3 s, the load is considered to be 500W. Similarly, the power output for the second period, which lasted from 0.3 to 0.6 s, was 750 watts, while the power output for the third period, which lasted from 0.6 to 0.9 s, was 1000 watts.

**Figure 16:**
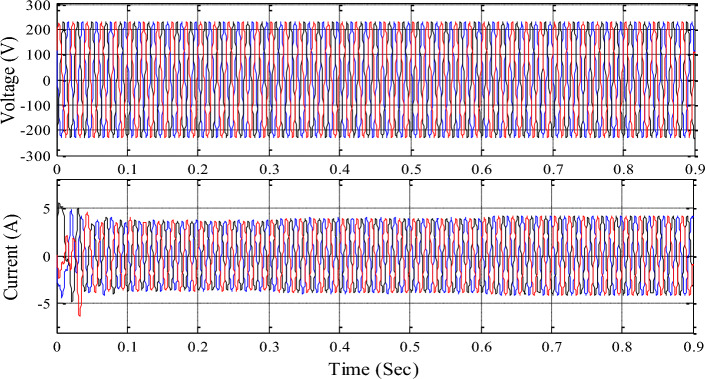
Inverter side voltage and current profile.

**Figure 17:**
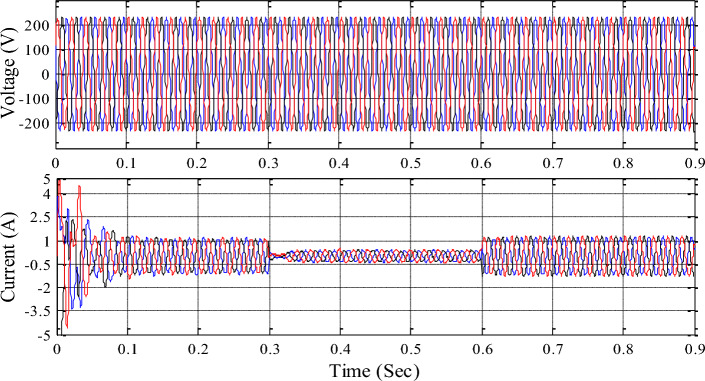
Grid voltage and current profile.

**Figure 18:**
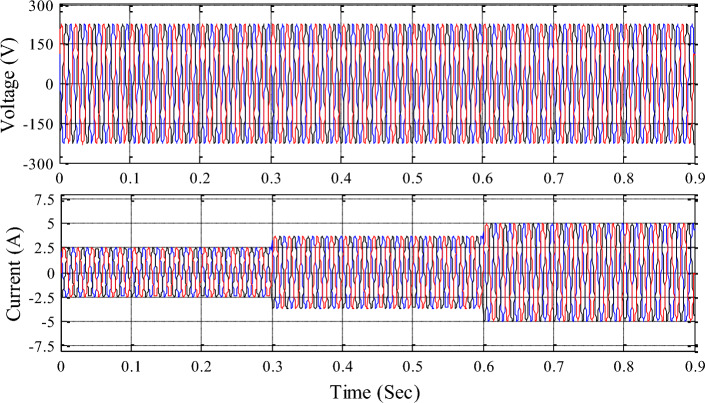
Load voltage and current profile.

The 3-phase voltage source inverter receives the common DC link voltage based on input source information and delivers sinusoidal pulse width modulation pulses to control it. Figures 16, 17, 18 show inverter, grid, and load voltages and currents.

**Figure 19:**
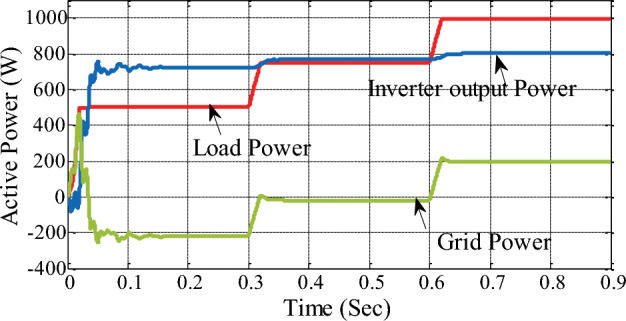
Active load, inverter and grid power profile.

In the proposed hybrid system, the load requirement is met by allocation with the grid based on the inverter output power, as shown in Fig. 19 for three different load scenarios. From zero to thirty seconds, the net inverter output is 754.1 W, the load mandate is 500 W, and the surplus of 254.1 W is fed back into the grid. Similarly, between 0.3 and 0.6 s, the load mandate is 750 W while the generated power is 780.4 W; the surplus 30.4 W power is fed to the grid; and between 0.6 and 0.9 s, the load mandate is 1000 W while the generated power is 802.2 W; the remaining 197.8 W power is drawn from the grid to meet the demand on the load side. The peak active power load demand from both the grid and the hybrid system is shown in Table 6.

**Table 6:** Summary of load demand with inverter output and grid power.

	Active power (W)		
Period (s)	0 to 0.3	0.3 to 0.6	0.6 to 0.9
Inverter power (W)	754.1	780.4	802.2
Grid (W)	− 254.1 W	− 30.4 W	197.8 W
Load (W)	500 W	750 W	1000 W

## Conclusion

The unified P&O and unified RBFN MPPT controllers are suggested in this work in conjunction with a hybrid Luo converter to build a hybrid RES system. The literature on hybrid energy sources that are sustainable covers a wide range of multi-input DC-DC converters and MPPT methods. A system that is hybrid has been proposed and researched by taking into account both the wind system (500 W) and the PV system (560 W). Taking into account data from an assortment of renewable sources throughout three separate load scenarios in three geographically varied areas, this study examines the built hybrid system's performance both off-grid and in grid-connected modes. The designed system's output is validated by simulation and hardware prototype model findings. In a stand-alone condition, the hybrid system generates an average of 756.7 W using a unified RBFN MPPT controller in the first region with a wind of 12 m/s and PV of 600 W/m^2^, 781.2 W in the second region with a wind of 10 m/s and 800 W/m^2^, and 804.6 W in the third region with an 8 m/s wind and 1000 W/m^2^. Correlations between the obtained findings and the P&O-MPPT controller confirm that the anticipated RBFN-MPPT controller offers the most promising option. This study presents the active powers profile for three distinct loads to verify the proposed controller's operation in a grid-tied setting. The complexity of implementing a hybrid system was reduced thanks to a new MPPT controller based on a modified RBFN.

## Data Availability

The datasets used and/or analysed during the current study are available from the corresponding author on reasonable request.
